# Storytelling in Medical Education: Narrative Medicine as a Resource for Interdisciplinary Collaboration

**DOI:** 10.3390/ijerph17041135

**Published:** 2020-02-11

**Authors:** Hung-Chang Liao, Ya-huei Wang

**Affiliations:** 1 Department of Health Services Administration, Chung Shan Medical University, Taichung 402, Taiwan; hcliao@csmu.edu.tw; 2 Department of Medical Education, Chung Shan Medical University Hospital, Taichung 402, Taiwan; 3 Department of Applied Foreign Languages, Chung Shan Medical University, Taichung 402, Taiwan

**Keywords:** narrative medicine, interdisciplinary collaboration, empathy, reflective thinking, patient–healthcare provider communication

## Abstract

*Objective:* The study intended to use narrative medicine study for interdisciplinary collaboration to let medical and healthcare students have a chance to interact with one another and listen to patients’ stories to enhance students’ reflective thinking, communication, empathy, and narrative medicine writing skills. *Methods:* A fifteen-week quasi-experimental design was used to examine the learning outcomes of the intervention. Two groups of students were randomly assigned as the experimental group (33 students) and the control group (32 students). Before and after the intervention, both groups had to fill in a Reflective Thinking Scale for Healthcare Students and Providers (RTS-HSP), Patient–Healthcare Provider Communication Scale (P-HCS), Empathy Scale in Patient Care (ES-PC), and Analytic Narrative Medicine Writing Scoring Rubric (ANMWSR). *Results:* The findings showed that on the reflective thinking scale, experimental group students had significantly higher reflective thinking posttest scores in “reflective skepticism,” “empathetic reflection,” and “critical open-mindedness,” but not in “self-examination.” As for patient–healthcare provider communication, they had significantly higher posttest scores in all “perception of trust and receptivity,” “patient-centered information giving,” “rapport building,” and “facilitation of patient involvement” factors. As for empathy, they had significant higher posttest scores in “behavioral empathy” and “affective empathy,” but not in “intelligent empathy.” In narrative medical writing skills, they had significant higher posttest scores in the “attention → representation → affiliation,” “depth of reflection,” “focus and context structure,” and “ideas and elaboration” sections, but not in the “language and conventions” section. *Conclusion:* The findings suggest that narrative medicine is worth recommending for interdisciplinary collaboration for healthcare education.

## 1. Introduction

Overreliance on medical technologies results in an inhumane medical care system, causing healthcare professionals not to care much about the feelings and psychological experiences of patients. Patients, of course, prefer to have more interaction with healthcare professionals than impersonal technologies [1]. However, healthcare professionals tend to view patients’ medical histories from a scientific perspective, not keeping in mind patients’ personal experiences. To improve the relationship between patients and healthcare professionals, since the 1980s, American medical schools and hospitals have promoted the humanities and, more recently, so-called narrative medicine in healthcare curricula and practices. These schools hope to facilitate practitioners’ reflection, thereby minimizing the distance between healthcare providers’ professional knowledge about diseases and patients’ subjective experiences with illnesses [2,3].

### 1.1. Narrative Medicine and Literature Study

According to Charon [4], narrative medicine is the medicine “practiced with the narrative competence to recognize, absorb, interpret, and be moved by the stories of illness” (p. 3). Research has shown that with the increase of clinical or healthcare training and practice, there may be a phenomenon of ethical erosion. Healthcare providers may lose an empathetic connection with patients and patients’ families [5,6]. In order to address the phenomenon of ethical erosion, narrative medicine can be used as an innovative tool to stimulate healthcare students’ and providers’ professionalism by letting them approach illness narratives with more empathetic connection and understanding [7]. It is medicine that uses narrative competence in diagnostic encounters and therapeutic processes to arouse empathy and understanding among physician, patients, patients’ families, therapists, medical technologists, and other healthcare professionals [8,9].

Providers who integrate narrative medicine into their practices can use self-reflection to examine their positions as physicians or professionals, endeavoring to research scientific objectivity to relieve patients’ pain and suffering [9]. Charon [4] declared that using narrative knowledge in the study of literature can facilitate empathy and reflection. Moreover, through literature study, readers can use other people’s experiences, which they may not want to personally experience, to learn life lessons [10,11,12].

Literature and narrative medicine share some similarities. Literature deals with suffering and dilemmas of all human beings; narrative medicine mainly deals with the suffering and dilemmas of patients, patients’ families, or healthcare practitioners [13]. Therefore, the narrative reading techniques used in literature study can also be used in narrative medicine study to better understand patients’ suffering and hence help medical and healthcare students and professionals build connections with patients [14,15].

### 1.2. Reflective Thinking

Both literature and narrative medicine are the sharing and reflection of human experiences, particularly regarding suffering, dilemmas, desperation, or even dying [4]. In “Literature and medicine,” Downie [16] suggests that literature can not only have entertainment value but also potentially provide insight into the complexity of varied professions and human experiences.

Research [17,18,19] has shown that when students and practitioners reflect, they may increase their capacities to consider situations from multiple perspectives in order to form empathic responses. Moreover, while deliberating on the dilemmatic situations regarding morality in literature, they may come to a sympathetic understanding of the human being, thus be more willing to accept the complexity of the lives for which they battle [20].

Narrative knowledge in literature study helps readers, through reflection, identify with others [21]. Therefore, through the study of literature and narrative medicine, students can use reflective thinking to examine people and situations from multiples angles to reach deeper understandings of human experiences. Those willing to consider different perspectives to manage personal and professional conflicts are more willing to collaborate with those with different disciplinary backgrounds in order to find optimal solutions to the healthcare problems.

### 1.3. Interdisciplinary Collaboration

To facilitate healthcare communication among students and professionals, teachers may group learners considering their disciplines, thereby creating an interdisciplinary collaborative learning class. In the interdisciplinary collaborative learning class, students with varied healthcare background knowledge, skills, and expertise can work together collaboratively to consider patients’ illnesses from different disciplinary perspectives [22].

The use of narrative medicine for interdisciplinary collaboration helps each participant actively take responsibility to work with one another to reach the set goal [23]. Moreover, it can help students and professionals read illness narratives from different perspectives, hence facilitating interpersonal relationships and understanding among physician, patients, patients’ families, and other healthcare professionals [24]. Thus, while making clinical/healthcare decisions, healthcare professionals can be more reflective and empathetic with patients’ or other healthcare professionals’ suffering and predicaments, hence building an affiliation with them and helping them reach decisions that favor their patients [25]. Moreover, through interdisciplinary collaboration and discussion from different perspectives, group members may share information, understand each member’s job responsibility, and thus develop more humanizing healthcare [26].

The aim of the research was to use narrative medicine for interdisciplinary collaboration to let students from different disciplines listen to illness narratives from different perspectives, through narrative medicine. To reach the goal, the study intended to examine the effectiveness of using narrative medicine for interdisciplinary collaboration in reflective thinking, patient–health provider communication, empathy, and narrative medicine writing skills. The proposed hypotheses were:

**Hypothesis** **1.** *Medical and healthcare students using narrative medicine for interdisciplinary collaboration will have higher reflective thinking levels than those not using narrative medicine*.

**Hypothesis** **2.** *Medical and healthcare students using narrative medicine for interdisciplinary collaboration will have better patient–healthcare provider communication than those not using narrative medicine*.

**Hypothesis** **3.** *Medical and healthcare students using narrative medicine for interdisciplinary collaboration will show more empathy than those not using narrative medicine*.

**Hypothesis** **4.** *Medical and healthcare students using narrative medicine for interdisciplinary collaboration will have higher levels of narrative medical writing skills than those not using narrative medicine*.

## 2. Materials and Methods

### 2.1. Participants

Two homogeneous and normally distributed classes that varied in medical and healthcare majors were randomly assigned as the experimental group (33 students) and the control group (32 students). Before participating in the research, all students (mean age = 19.12; *SD* = 0.39) were informed the research purposes to let them decide whether to participate in the study. However, in order to decrease the unexpected outcome as confounding effects, such as Hawthorne effect or John Henry effect, the students did not know which groups there were in. All 65 students were college students with varied majors related to medical and healthcare disciplines, such as medical laboratory and biotechnology, speech language pathology and audiology, medical imaging and radiological sciences, psychology, occupational therapy, physical therapy, medicine, dentistry, nursing, etc. They were first-year and second-year college students, hence having little or no clinical experience. In addition, with different majors, both group students had little or almost no interaction outside the class. The research was approved by Institutional Review Board of the Chung Shang Medical University Hospital (CSMUH No. 14149). The participants were informed about the research purpose. All data from participants were blinded and all data were identified using numbers in order to prevent bringing any potential harm to students.

### 2.2. Experimental Design

A quasi-experimental design was conducted to test the possibility of using narrative medicine for interdisciplinary collaboration in terms of reflective thinking, patient–healthcare provider communication, empathy, and narrative medicine writing skills. Before and after the 15-week intervention, a Reflective Thinking Scale for Healthcare Students and Providers (RTS-HSP), a Patient–Healthcare Provider Communication Scale (P-HCS), an Empathy Scale in Patient Care (ES-PC), and an Analytic Narrative Medicine Writing Scoring Rubric (ANMWSR) were administered to both groups, and the researchers collected, analyzed, and compared the test results. Based on these learning outcomes, the researchers used the cluster algorithm for interdisciplinary collaboration to evaluate the efficacy of study of literature for the experimental group in comparison with the control group. Both the experimental group and control group used techniques for interdisciplinary collaboration to investigate ethical or moral dilemmas in stories, novels, dramas, or films. However, the experimental group used ideas from narrative medicine for purposes of such collaborative study, while the control group did not. After intervention, the participants’ posttest results of RTS-HSP, P-HCS, ES-PC, and ANMWSR were compared to evaluate learning performances.

### 2.3. Cluster Algorithm for Interdisciplinary Collaboration

In reference to Liao and Wang’s [27] cluster algorithms for heterogeneous and non-heterogeneous cluster grouping, the researchers adapted the algorithms and used the five variables—gender, major, RTS-HSP, P-HCS, and ES-PC—as the variables for interdisciplinary collaboration, based on the following process. First, the researchers collected the pretest scores of the RTS-HSP, P-HCS, and ES-PC, and to avoid different measurement standards at different scale levels, each student’s pretest scores of RTS-HSP, P-HCS, and ES-PC were transformed into ratio scores. For example, if the student gets a score of 122 in RTS-HSP, and in the pretest the score range for all students is between 96 and 174, the ratio score would be 0.333, that is $\frac{122-96}{174-96}$. After obtaining all students’ ratio scores of RTS-HSP, P-HCS, and ES-PC, the researchers calculated the diverse effect to arrange the interdisciplinary learning clusters, with the criterion set as $\left|w_{j}\sum_{i,j}^{c}R_{i,j}\right|\leq \theta$, where $w_{j}$ was the fuzzy weights ($\sum_{j=1}^{3}w_{j}=1$), *c* was the number of students in the cluster, *i* was the *i*th student in the cluster, *j* was the ratio score in the RTS-HSP, P-HCS, and ES-PC, and θ was the threshold. $R_{i,j}$ was the distance and could be computed as “the ratio score minus the mean of ratio score in the cluster.” When the criterion is used, one key point must be reminded that in each learning cluster there should be no less than two females and two males and should be with different majors. Table 1 shows the derived interdisciplinary collaborative learning clusters for both groups.

### 2.4. Procedure

**Figure 1:**
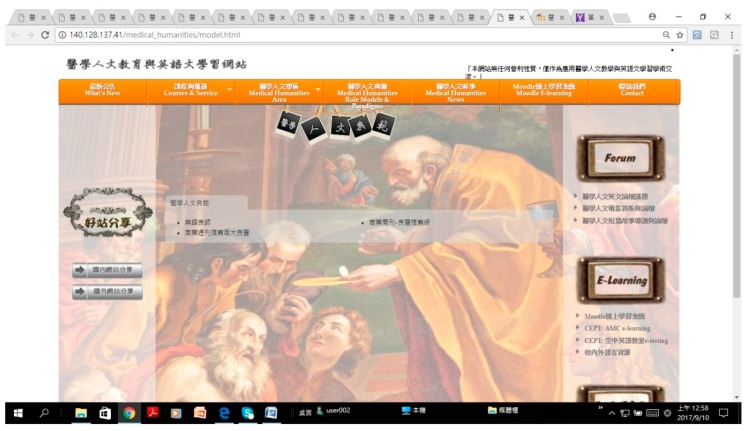
E-learning learning website for discussion forum.

The experiment lasted for 15 weeks. Both experimental and control group students attended two-hour class instruction per week and at least two hours of independent study and discussion forum each week. Both the experimental group and control group used techniques for interdisciplinary collaboration to investigate ethical or moral dilemmas in stories, novels, dramas, or films. In addition, they had to go to the E-learning learning website for discussion forum (Figure 1) for independent and interdisciplinary collaboration, according to the proposed content and topics in literature study schedule (see Table 2). Students were required to go to the discussion forum in the website to post their reflections. In addition, they were encouraged to bring discussion topics they wanted to share with classmates. The only difference between the two groups was that only the experimental group used ideas from narrative medicine for purposes of such collaborative study, while the control group did not.

More specifically, the experimental-group students were taught the fundamental stages of narrative medicine, as characterized by Rita Charon [4], to strengthen the students’ narrative competence and enhance their sense of how stories can foster empathetic connection to others’ suffering. The fundamental stages of narrative medicine identified by Charon are: attention, representation, and affiliation [4]. In the attention stage, students were encouraged to be mindfully attentive in clinical encounters, paying close attention to patients’ predicaments, conflicts, or dilemmas. In the representation stage, students were encouraged to engage actively in such clinical encounters, predicaments, conflicts, and dilemmas, so as to exercise their ethical/moral imagination, representing what they encounter so that they understood it anew. In the affiliation stage, students were encouraged to build on the prior stages of attention and representation in order to develop the capacity for affiliation—that is, the capacity to be emotionally connected with patients or those suffering alongside them. After being guided through each of these fundamental stages, students in the experimental group were also required to use the triad of attention → representation → affiliation as a lens for literary study. During this phase, they used literary works to realize and reflect upon, from different perspectives, what is at stake in human illness or suffering, with a view to becoming more empathetic and reflective vis-à-vis the dilemmas and conflict-ridden situations associated with ill health.

### 2.5. Measures, Validity, and Reliability

#### 2.5.1. Reflective Thinking Scale for Healthcare Students and Providers (RTS-HSP)

In order to measure students’ reflective thinking levels, the study used a 22-item Reflective Thinking Scale for Healthcare Students and Providers (RTS-HSP) [37]. The scale was on a nine-point Likert scale, from 9 (always) to 1 (never). Exploratory factor analysis had been used to test the construct validity and internal consistency reliability of the RTS-HSP [37]. The final analysis yielded four factors, accounting for 56.66% of the total variance: reflective skepticism (six items), self-examination (six items), empathetic reflection (five items), and critical open-mindedness (five items). The Cronbach’s alphas for the entire scale and four subscales were 0.87, 0.84, 0.84, 0.80, and 0.77. The test–retest correlation coefficients were 0.82, 0.78, 0.74, 0.73, and 0.84, indicating appropriate stability and reliability of the scale.

#### 2.5.2. Patient–Healthcare Provider Communication Scale (P-HCS)

A 26-item, nine-point Likert scale Patient–Healthcare Provider Communication Scale (P-HCS) [38] was used to measure the communication levels, with 9 meaning “strongly important” and 1 meaning “strongly not important.” The validity and reliability of the P–HCS instrument had been tested using exploratory factor analysis and reliability analysis [38]. The final analysis yielded four factors, accounting for 57.08% of the total variance: perception of trust and receptivity (seven items), patient-centered information giving (six items), rapport building (seven items), and facilitation of patient involvement (six items). The Cronbach’s alphas for the entire scale and four subscales were 0.93, 0.89, 0.82, 0.83, and 0.72. The test–retest correlation coefficients were 0.84, 0.80, 0.85, 0.81, and 0.87.

#### 2.5.3. Empathy Scale in Patient Care (ES-PC)

The Empathy Scale in Patient Care [39] was used to test students’ empathy levels. The ES-PC is a 23-item, nine-point Likert scale, with 9 meaning “strongly agree” to 1 meaning “strongly disagree.” Exploratory factor analysis had been used to test the construct validity and internal consistency reliability of the ES-PC [39]. The final analysis yielded three factors, accounting for 61.43 of the total variance: behavioral empathy (nine items); affective empathy (seven items), and intellectual empathy (seven items). Behavioral empathy refers to the capacity to use observable gestures to reveal ones’ understanding of patients’ suffering or feelings. Affective empathy refers to the capacity to affectively feel the suffering of patients. Intellectual empathy refers to the capacity to consciously put oneself in the place of patients and see things from their perspectives without giving any criticism. The Cronbach’s alphas for the entire and three subscales were 0.94, 0.93, 0.87, and 0.88.

#### 2.5.4. Analytic Narrative Medicine Writing Scoring Rubric (ANMWSR)

To assess students’ levels of narrative medicine writing, an Analytic Narrative Medicine Writing Scoring Rubric [40] had been developed and tested to assess students’ narrative medicine writing skills in “attention → representation → affiliation,” “depth of reflection,” “focus & context structure,” “ideas & elaboration,” and “language & conventions,” using a 0- to 5-point rating. The researchers used Item Objective Congruence (IOC) Index to determine the content validity of ANMWSR. The developed ANMWSR reached the acceptable IOC indexes, ranging from 0.5 to 1.0. The consensus estimates of inter-rater reliability with percent exact and adjacent agreements were between 92% and 100%. The consistent estimates of inter-rater reliabilities were between 0.81 and 0.88. Intra-rater reliability was 0.92 and 0.99.

### 2.6. Data Analysis

The researchers used SPSS 14.0 (SPSS Inc., Chicago, IL, USA) to analyze quantitative data, including one-way MANOVA (Multivariate Analysis of Variance) to compare multivariate sample means and one-way MANCOVA (Multivariate Analysis of Covariance), with pretest scores as covariates to control for the initial group differences. The confidence level was set at 95% (*p* < 0.05).

## 3. Results

**Hypothesis** **1.** *Medical and healthcare students using narrative medicine for interdisciplinary collaboration will have higher reflective thinking level than those not using narrative medicine*.

To test Hypothesis 1, using one-way MANOVA, the pretest results showed no overall significant difference (Wilks’ Lambda: 0.963; *F* (4, 60) = 0.574; *p* = 0.682) between the experimental and control groups. In addition, the pretest results also showed no significant differences between the experimental group (Means = 31.97, 35.70, 29.12, and 32.28) and the control group (Means = 30.00, 34.16, 28.75, and 30.41) on the reflective skepticism (*F* (1, 63) = 1.386; *p* = 0.244), self-examination (*F* (1, 63) = 0.709; *p* = 0.403), empathetic reflection (*F* (1, 63) = 0.066; *p* = 0.798), and critical open-mindedness (*F* (1, 63) = 1.209; *p* = 0.276).

The researchers, after intervention, used one-way MANCOVA to examine whether reflective thinking pretest performances would bring any difference to the posttest performances, with the pretest results as covariates. The MANCOVA results revealed no significant relatedness in the empathetic reflection pretest and posttest performance (Wilks’ Lambda: 0.849; *F* (4, 56) = 2.497; *p* = 0.053 > 0.05). However, a significant relatedness existed between the pretests and posttests in “reflective skepticism” (Wilks’ Lambda: 0.766; *F* (4, 56) = 4.277; *p* = 0.004; *p* < 0.01), “self-examination” (Wilks’ Lambda: 0.677; *F* (4, 56) = 6.689; *p* < 0.000), and “critical open-mindedness” (Wilks’ Lambda: 0.693; *F* (4, 56) = 6.204; *p* < 0.000) sections (see Table 3).

While looking into the adjusted posttest means (see Table 4), the researchers found that in the “reflective skepticism,” “empathetic reflection,” and “critical open-mindedness,” the experimental group students had significantly higher scores (Means = 46.35, 40.79, and 40.51) than the control group (Means = 42.51, 37.94; *p* < 0.05 and 36.71; *p* < 0.01). Nonetheless, in the “self-examination” factor, the experimental group students had a higher score (Mean = 46.64) than control group (Mean = 44.68), but not reaching significant difference (*p = 0*.121 > 0.05).

**Hypothesis** **2.** *Medical and healthcare students using narrative medicine for interdisciplinary collaboration will have better patient–healthcare provider communication than those not using narrative medicine*.

To test Hypothesis 2, with the use of one-way MANOVA, the pretest results revealed no overall significant differences (Wilks’ Lambda: 0.956; *F* (4, 60) = 0.697; *p* = 0.597) between the two groups. In addition, the results showed no significant differences between the means of the groups (Experimental Group: Means = 47.15, 43.76, 48.33, and 41.61; Control Group: Means = 50.22, 45.72, 50.78, and 42.81) on the “perception of trust and receptivity”(*F* (1, 63) = 2.183; *p* = 0.145), “patient-centered information giving” (*F* (1, 63) = 1.231; *p* = 0.271), “rapport building” (*F* (1, 63) = 1.342; *p* = 0.251), and “facilitation of patient involvement” (*F* (1, 63) = 0.355; *p* = 0.553).

After 15-week intervention, the one-way MANCOVA results presented a significant relatedness between the pretest and posttest performances in “perception of trust and receptivity (Wilks’ Lambda: 0.583; *F* (4, 56) = 10.019; *p* < 0.000), “patient-centered information giving” (Wilks’ Lambda: 0.700; *F* (4, 56) = 5.990; *p* < 0.000), “rapport building” (Wilks’ Lambda: 0.344; *F* (4, 56) = 26.708; *p* < 0.000), and “facilitation of patient involvement” (Wilks’ Lambda: 0.620; *F* (4, 56) = 8.580; *p* < 0.000; see Table 5).

While looking into the adjusted posttest means, the MANCOVA results showed that the experimental group had significantly higher adjusted posttest means (Means = 58.96, 52.34, 59.14, and 51.71) than the means of the control group (Means = 55.35, 48.06, 54.04, and 46.80; *p* < 0.000; see Table 6).

**Hypothesis** **3.** *Medical and healthcare students using narrative medicine for interdisciplinary collaboration will show more empathy than those not using narrative medicine*.

The pretest results showed that, via one-way MANOVA, there was no overall significant difference (Wilks’ Lambda: 0.975; *F* (3, 61) = 0.524; *p* = 0.667) between the two groups. In addition, the results presented no significant differences between the means of the experimental group (Means = 56.00, 39.92, and 41.14) and the means of the control group (Means = 58.72, 41.84, and 42.63) in “behavioral empathy” (*F* (1, 63) = 1.293; *p* = 0.260), “affective empathy” (*F* (1, 63) = 0.998; *p* = 0.322), and “intellectual empathy” (*F* (1, 63) = 0.514; *p* = 0.465).

After 15-week intervention, the results showed no significant relatedness between the pretest and posttest performances in “behavioral empathy” (Wilks’ Lambda: 0.906; *F* (3, 58) = 2.006; *p* = 0.123 > 0.05) and “affective empathy” (Wilks’ Lambda: 0.924; *F* (3, 58) = 1.582; *p* = 0.203 > 0.05). However, the results showed a significant relatedness between the pretest and posttest performance in “intellectual empathy” (Wilks’ Lambda: 0.816; *F* (3, 58) = 4.366; *p* = 0.008 < 0.01; see Table 7).

While looking into the adjusted posttest means of the “behavioral empathy” and “affective empathy,” the MANCOVA results showed that there were significant differences in the adjusted means between the experimental group (Means = 74.14 and 51.91) and the control group (Means = 68.49 and 44.74; *p* < 0.01). Nonetheless, there was no significant difference between two groups in “intellectual empathy” (Experimental Group: Mean = 54.26; Control Group: Mean = 52.44; *p* = 0.063 > 0.05; see Table 8).

**Hypothesis** **4.** *Medical and healthcare students using narrative medicine for interdisciplinary collaboration will have higher levels of narrative medical writing skills than those not using narrative medicine*.

The pretest results showed that via one-way MANOVA there was no overall significant difference (Wilks’ Lambda: 0.944; *F* (5, 59) = 0.695; *p* = 0.629) between the two groups. In addition, the pretest results showed no significant differences between the experimental group (Means = 2.24, 2.48, 2.79, 2.70, and 3.30) and the control group (Means = 2.16, 2.66, 2.91, 2.75, and 3.38) in “attention → representation → affiliation” (*F* (1, 63) = 0.532; *p* = 0.468), “depth of reflection” (*F* (1, 63) = 1.945; *p* = 0.168), “focus & context structure” (*F* (1, 63) = 0.787; *p* = 0.379), “ideas & elaboration” (*F* (1, 63) = 0.170; *p* = 0.682), and “language & conventions” (*F* (1, 63) = 0.174; *p* = 0.678).

After 15-week intervention, the one-way MANCOVA results showed no significant relatedness between the pretest and posttest performances in “depth of reflection” (Wilks’ Lambda: 0.901; *F* (5, 54) = 1.190; *p* = 0.326 > 0.05), “focus & context structure” (Wilks’ Lambda: 0.843; *F* (5, 54) = 2.011; *p* = 0.092 > 0.05), “ideas & elaboration” (Wilks’ Lambda: 0.954; *F* (5, 54) = 0.520; *p* = 0.760 > 0.05), and “language & conventions” (Wilks’ Lambda: 0.953; *F* (5, 54) = 0.531; *p* = 0.752 > 0.05). However, there was a significant relatedness between the pretest and posttest in “attention → representation → affiliation” (Wilks’ Lambda: 0.812; *F* (5, 54) = 2.503; *p* = 0.041 < 0.05; see Table 9).

While looking into the adjusted posttest means of the factors, the MANCOVA results showed that the experimental group had significantly higher adjusted posttest means (Means = 4.33, 4.04, 4.52, and 4.08) than the means of the control group (Means = 2.94 and 3.21, *p* < 0.000; 4.18, and 3.76; *p* < 0.05). Nonetheless, in “language and conventions,” there was no significant difference between these two groups (Experimental Group: Mean = 4.09; Control Group: Mean = 3.88; *p* = 0.094 > 0.05; See Table 10).

## 4. Discussion

The study used narrative medicine for interdisciplinary collaboration to strengthen medical and healthcare students’ reflective thinking, patient–healthcare provider communication, empathy, and narrative medicine writing skills. The results reveal that after 15-week intervention, both group students get higher performances in reflective thinking, patient–healthcare provider communication, empathy, and narrative medicine writing skills. The research results thus suggest that interdisciplinary collaboration can have positive effects on students whether or not they are situated in narrative medicine. In addition, students situated in narrative medicine have higher performances in reflective thinking, patient–healthcare provider communication, empathy, and narrative medicine writing skills, though some areas do not show significant differences. Regarding reflective thinking, students using narrative medicine for interdisciplinary collaboration had significantly higher reflective thinking levels than those who did not use narrative medicine in “reflective skepticism,” “empathetic reflection,” and “critical open-mindedness.” The results imply that students who received narrative medicine study have more chances, listening to abundant illness stories, to discover the dilemmas patients, patients’ families, and healthcare professionals encounter. These illness stories also give students more time to reflect on widely accepted knowledge. Hence, in order to lessen patients’ suffering, students could justify patients’ situations and check the credibility of information sources to find optimal solutions for patients and their families. The results correspond with Karkabi, Wald, and Castel’s [41] study and Charon’s [4] study in that the combination of literature study and narratives can foster reflection, because they can train participants to become active listeners, sharing illness narrative reading and interacting with multiple perspectives and texts.

However, the results showed no significant difference in the “self-examination” section, though the students who received narrative medicine study had higher scores than those who did not receive the study. It could be possible that students are still too young to use self-examination without impartial analysis to decipher what their behaviors, thoughts, and feelings mean [42].

As for patient–healthcare provider communication, students using narrative medicine had significantly higher patient–healthcare provider communication than those not using narrative medicine in all “perception of trust and receptivity,” “patient-centered information giving,” “rapport building,” and “facilitation of patient involvement” factors. The results imply that students receiving narrative medicine study can use the narrative medicine techniques, such as attention→ representation → affiliation, to empathize with patients’ inner and psychological perspectives. Therefore, in rapport with patients, students are more willing to offer patient-centered information, involving patients in their own healthcare decision-making, thus maintaining smoother and more harmonious communication with them. The results correspond with Hojat, et al.’s [24] study, because narrative knowledge can serve as a vehicle to fill in communication problems, leading to better interpersonal relationships with patients, patients’ families, and healthcare professionals.

Students who received narrative medicine study also showed more empathy than those who did not receive the study in the “behavioral empathy” and “affective empathy” section. The results imply that students situated in narrative medicine study are more willing to listen to patients, using facial expressions and eye contact to make them feel respected, and are inclined to comfort patients and patients’ families, if needed. They may also feel sad when they see patients’ misfortune and suffering. The results correspond with Charon’s [43,44] studies that showed that from conversations with patients, healthcare professionals followed the narrative threads of the stories to derive complicated illness narratives, imagining the mental, familial, cultural, or economic situations patients were in. As they enter into patients’ illness narratives and experience their pain and fear, healthcare professionals can experience and extend empathy for those who suffer, and further stand with patients to deal with their illnesses.

However, the analysis results showed no significant difference in “intelligent empathy.” It could be that intelligent empathy involves consciously putting oneself in the place of patients and seeing things from their perspectives without any criticism to understand their emotions and consequences [45]. Hence, in order to reach a higher level of empathetic recognition, students need more time to practice reconstructing the viewpoints of patients to themselves to become impartial observers.

In addition, students receiving narrative medicine study reached a higher level of narrative medical writing skills than those who did not in the “attention → representation → affiliation,” “depth of reflection,” “focus and context structure,” and “ideas and elaboration” sections. The results imply that students receiving narrative medicine study can use the triad of “attention → representation → affiliation” and connect their personal feelings to write touching illness stories. Moreover, these students can analyze and contemplate patients’ situations from different perspectives to arouse readers’ empathy toward patients’ dilemmas and suffering. However, there was no significance in the “language and conventions” section. It could be because that “language and conventions” are the basic feature of effective writing communication, or because students in the study are medical university students, with above-average college entrance scores. Therefore, these students have less difficulty with spelling, punctuation, and sentence structure.

## 5. Conclusions

The study proposed using narrative medicine for interdisciplinary collaboration to let medical and healthcare students from different disciplines interact with one another and listen to illness narratives from multiple perspectives to realize people’s dilemmas and suffering, further building connections with patients’ illnesses. The results show that the use of narrative medicine can have positive effects on students in self-reflective thinking, in patient–healthcare provider communication, in empathy, and in narrative medicine writing skills, though not showing significant differences in some subscales.

The study intended to allow medical and healthcare students in different disciplines listen to diverse voices in illness narratives and let them reflect, empathize, and communicate with patients. The contribution of the study lies in that students with different disciplines can acquire and share narrative reading and writing with one another, using narrative medicine techniques, such as attention → representation → affiliation, to reflect upon illness narratives from multiple perspectives. Hence, they can realize people’s suffering and dilemmas, learn to empathize and communicate with patients, and care about patients’ inner worlds. There is another contribution in the development of interdisciplinary collaborative learning, clusters, without which a successful implementation of narrative medicine for interdisciplinary collaboration in the study would not have been possible.

The study demonstrates a successful attempt to use narrative medicine for interdisciplinary collaboration. However, continued cross-disciplinary and interdisciplinary collaboration is needed to further verify the study. Future study may consider the interrelations among communication, reflective thinking, empathy, and narrative medicine writing levels.

## Figures and Tables

**Table 1:** The derived interdisciplinary collaborative learning clusters.

	Group	Experimental Group	Control Group
Cluster Number			
1		5, 15, 17. 21	4, 14, 15, 21
2		7, 9, 23, 25	9, 16, 22, 25
3		3, 12, 19, 22	6, 7, 12, 17
4		4, 8, 10, 27	3, 20, 27, 30
5		6, 16, 24, 28	1, 11, 19, 26
6		2, 13, 31, 33,	10, 18, 23, 28
7		1, 14, 18, 20	5, 8, 24, 31
8		11, 26, 29, 30, 32	2, 13, 29, 32

**Table 2:** Course content and discussion topics.

Week	Course Content and Discussion Topics
1–2	Keywords & Lecturing: medical humanities; empathy versus compassion; professionalism; medical professionalism; narrations and story telling Teaching Content: introduction to medical humanities; literature and medicine; medicine as art or as science; How can art and humanities help facilitate communication, understanding, and empathy? Literature Study: “Heart-wrenching photo of doctor crying goes viral. Here’s why” [28] Conflicts & Dilemmas: Do you think healthcare professionals should show appropriate compassion and empathy towards patients? Can doctors cry? Why or why not?
3–5	Keywords & Lecturing: terminal cancer; DNR (Do Not Resuscitate) option; dying with dignity; human interaction; doctor–patient relationship; medical team; human beings versus research objects Literature Study: *Wit: A play* [29]; *Wit* [30] Conflicts & Dilemmas: Do you think patients have the right to choose the DNR option once they are ready to die? Do you think only God alone has the right to terminate life? Why or why not?
6–7	Keywords & Lecturing: rest cure versus writing cure; doctor husband versus doctor’s wife; female voice; marginalized community; narrators; madness Literature Study: *The Yellow Wallpaper* [31] Conflicts & Dilemmas: Do you think it is better to go on a rest cure or writing cure after childbirth? Do you think women (women, or both genders) are oppressed under the patriarchal society? Are you willing to marry a doctor husband? Why or why not?
8–10	Keywords & Lecturing: Alzheimer’s disease; aging; physician husband; empathy versus detachment; self-killing/suicide; life value and dignity. Literature Study: *Still Alice* [32]; *Still Alice* [33] Conflicts & Dilemmas: In your opinion, what is the definition of “aging”? Do you think the government should make a policy to stop medical treatment for the aging and “useless eaters”?
11–12	Keywords & Lecturing: female subjectivity; soul freedom/release; meaning in life; identity recognition; depression and mental illness Literature Study: *To Room Nineteen* [34] Conflicts & Dilemmas: Do you think women should be financially independent or dependent upon men; Do you think it is morally acceptable to commit suicide if one decides life is meaningless or if one finds their heath is unrecoverable? Why or why not?
13–15	Keywords & Lecturing: trauma; domestic violence; sacrifice versus contribution; marginalized community; born good versus born evil Literature Study: *Pay It Forward* [35]; *Pay It Forward* [36] Conflicts & Dilemmas: Do you think people are basically good or evil? Do you want to “pay it forward”? Why or why not?

**Table 3:** Multivariate Analysis of Covariance (MANCOVA) results for the Reflective Thinking Scale for Healthcare Students and Providers (RTS-HSP).

Test	Covariance			
Posttest	Reflective skepticism (pretest)	Self-examination (pretest)	Empathetic reflection (pretest)	Critical open-mindedness (pretest)
	Wilk’s Λ *F* (4, 56) (*p*-value)			
	0.766 4.277 (0.004 **)	0.677 6.689 (<0.000 **)	0.849 2.497 (0.053)	0.693 6.204 (<0.000 **)
	Tests of Between-Subjects Effects *F* (1, 59) (*p*-value)			
Reflective skepticism	9.665 (0.003 **)	0.018 (0.895)	0.000 (0.988)	0.064 (0.801)
Self-examination	0.000 (0.990)	16.238 (<0.000 **)	0.153 (0.697)	0.103 (0.749)
Empathetic reflection	0.034 (0.854)	2.005 (0.162)	3.907 (0.053)	0.403 (0.528)
Critical open-mindedness	0.155 (0.695)	1.210 (0.276)	0.238 (0.628)	22.891 (<0.000 **)

Experimental: *N* = 33; Control: *N* = 32. Wilk’s Λ: Wilks Lambda. *F* (4, 56): *F* (hypothesis degrees of freedom, error degrees of freedom). ** *p* < 0.01.

**Table 4:** MANCOVA and the adjusted posttest means for the RTS-HSP.

Posttest	Group	Adjusted Mean	(SD)	*p*-Value
Reflective skepticism	Experimental Control	46.35 42.51	(1.08) (1.10)	0.016 *
Self-examination	Experimental Control	46.64 44.68	(0.87) (0.88)	0.121
Empathetic reflection	Experimental Control	40.79 37.94	(0.82) (0.83)	0.019 *
Critical open-mindedness	Experimental Control	40.51 36.71	(0.96) (0.98)	0.008 **

Experimental: *N* = 33; Control: *N* = 32. SD: Standard deviation. * *p* < 0.05, ** *p* < 0.01.

**Table 5:** MANCOVA results for the Patient–Healthcare Provider Communication Scale (P-HCS) posttest.

Test	Covariance			
Posttest	Perception of trust and receptivity (pretest)	Patient-centered information giving (pretest)	Rapport building (pretest)	Facilitation of patient involvement (pretest)
	Wilk’s Λ *F* (4, 56) (*p*-value)			
	0.583 10.019 (<0.000 **)	0.700 5.990 (<0.000 **)	0.344 26.708 (<0.000 **)	0.620 8.580 (<0.000 **)
	Tests of Between-Subjects Effects *F* (1, 59) (*p*-value)			
Perception of trust and receptivity	17.794 (<0.000 **)	0.973 (0.328)	5.601 (0.021 *)	1.107 (0.297)
Patient-centered information giving	2.002 (0.968)	7.846 (0.007 **)	0.802 (0.374)	0.284 (0.596)
Rapport building	0.368 (0.547)	0.464 (0.499)	78.664 (<0.000 **)	4.989 (0.029 *)
Facilitation of patient involvement	2.487 (0.488)	3.798 (0.056)	0.802 (0.374)	6.584 (0.013)

Experimental: *N* = 33; Control: *N* = 32. Wilk’s Λ: Wilks Lambda. *F* (4, 56): *F* hypothesis degrees of freedom, error degrees of freedom). * *p* < 0.05, ** *p* < 0.01.

**Table 6:** MANCOVA and the adjusted posttest means for the P-HCS.

Posttest	Group	Adjusted Mean	(SD)	*p*-Value
Perception of trust and receptivity	Experimental Control	58.96 55.35	(0.75) (0.76)	0.001 **
Patient-centered information giving	Experimental Control	52.34 48.06	(0.58) (0.58)	<0.000 **
Rapport building	Experimental Control	59.14 54.04	(0.70) (0.71)	<0.000 **
Facilitation of patient involvement	Experimental Control	51.71 46.80	(0.57) (0.58)	<0.000 **

Experimental: *N* = 33; Control: *N* = 32. SD: Standard deviation. ** *p <* 0.01.

**Table 7:** MANCOVA results for the Empathy Scale in Patient Care (ES-PC) posttest.

Test	Covariance		
Posttest	Behavioral empathy (pretest)	Affective empathy (pretest)	Intellectual empathy (pretest)
	Wilk’s Λ *F* (3, 58) (*p*-value)		
	0.906 2.006 (0.123)	0.924 1.582 (0.203)	0.816 4.366 (0.008 **)
	Tests of Between-Subjects Effects *F* (1, 60) (*p*-value)		
Behavioral empathy	3.888 (0.053)	0.184 (0.669)	0.627 (0.432)
Affective empathy	2.135 (0.149)	3.451 (0.068)	4.559 (0.037 *)
Intellectual empathy	2.228 (0. 141)	1.920 (0.171)	10.334 (0.002 **)

Experimental: *N* = 33; Control: *N* = 32. Wilk’s Λ: Wilks Lambda. *F* (3, 58): *F* (hypothesis degrees of freedom, error degrees of freedom). * *p* < 0.05, ** *p* < 0.01.

**Table 8:** MANCOVA and the adjusted posttest means for the ES-PC.

Posttest	Group	Adjusted Mean	(SD)	*p*-Value
Behavioral empathy	Experimental Control	74.14 68.49	(1.12) (1.14)	0.001 **
Affective empathy	Experimental Control	51.91 44.74	(1.37) (1.39)	0.001 **
Intellectual empathy	Experimental Control	54.26 52.44	(0.67) (0.68)	0.063

Experimental: *N* = 33; Control: *N* = 32. SD: Standard deviation. ** *p* < 0.01.

**Table 9:** MANCOVA results for the Analytic Narrative Medicine Writing Scoring Rubric (ANMWSR) posttest.

Test	Covariance				
Posttest	Attention → Representation → affiliation (pretest)	Depth of reflection (pretest)	Focus & context structure (pretest)	Ideas & elaboration (pretest)	Language & conventions (pretest)
	Wilk’s Λ *F* (5, 54) (*p*-value)				
	0.812 2.503 (0.041 *)	0.901 1.190 (0.326)	0.843 2.011 (0.092)	0.954 0.520 (0.760)	0.953 0.531 (0.752)
	Tests of Between-Subjects Effects *F* (1, 58) (*p*-value)				
Attention → representation → affiliation	2.424 (0.125)	4.255 (0.044 *)	4.088 (0.048 *)	1.661 (0.203)	0.134 (0.715)
Depth of reflection	1.931 (0.170)	0.003 (0.960)	1.266 (0.265)	0.494 (0.485)	0.291 (0.592)
Focus & context structure	6.266 (0.015 *)	1.083 (0.302)	0.114 (0.737)	0.067 (0.797)	2.479 (0.121)
Ideas & elaboration	0.354 (0.554)	0.029 (0.866)	0.554 (0.460)	0.058 (0.810)	0.004 (0.949)
Language & conventions	0.000 (0.999)	1.598 (0.211)	2.046 (0.158)	1.027 (0.315)	0.006 (0.938)

Experimental: *N* = 33; Control: *N* = 32. Wilk’s Λ: Wilks Lambda. *F* (5, 54): *F* (hypothesis degrees of freedom, error degrees of freedom). * *p* < 0.05.

**Table 10:** MANCOVA and the adjusted posttest means for the ANMWSR.

Posttest	Group	Mean	(SD)	*p*-Value
Attention → representation → affiliation	Experimental Control	4.33 2.94	(0.11) (0.11)	<0.000 **
Depth of reflection	Experimental Control	4.04 3.21	(0.10) (0.10)	<0.000 **
Focus & context structure	Experimental Control	4.52 4.18	(0.09) (0.09)	0.012 *
Ideas & elaboration	Experimental Control	4.08 3.76	(0.10) (0.11)	0.041 *
Language & conventions	Experimental Control	4.09 3.88	(0.09) (0.09)	0.094

Experimental: *N* = 33; Control: *N* = 32. SD: Standard deviation. * *p* < 0.05, ** *p* < 0.01.
